# The Combined Effects of an Anti‐Inflammatory Diet and Curcumin Supplementation on Thyroid Function and Lipid Profile in Patients With Hashimoto's Thyroiditis: A Double Blind Randomised Clinical Trial

**DOI:** 10.1002/edm2.70138

**Published:** 2025-12-02

**Authors:** Fatemeh Bourbour, Behnam Mahdavi, Niayesh Naghshi, Zahra Yari, Seyedsina Moghimnejad hosseini, Saeid Kalbasi, Golbon Sohrab

**Affiliations:** ^1^ Department of Clinical Nutrition and Dietetics, Faculty of Nutrition Sciences and Food Technology, National Nutrition and Food Technology Research Institute Shahid Beheshti University of Medical Sciences Tehran Iran; ^2^ Saeed Pathobiology and Genetics Lab Tehran Iran; ^3^ Department of Nutrition Research, National Nutrition and Food Technology Research Institute and Faculty of Nutrition Sciences and Food Technology Shahid Beheshti University of Medical Sciences Tehran Iran; ^4^ Faculty of Medicine Semmelweis University Budapest Hungary; ^5^ Internal Medicine Ward, Endocrinology and Metabolism Section Shahid Beheshti University of Medical Sciences Tehran Iran

**Keywords:** anti‐inflammatory diet, curcumin, Hashimoto's thyroiditis, lipid profile, thyroxine

## Abstract

**Background:**

Hashimoto's thyroiditis (HT) is an inflammatory autoimmune disease and patients with HT may benefit from interventions that incorporate anti‐inflammatory components. This study aimed to assess the combined effects of an anti‐inflammatory diet and curcumin supplementation on thyroid hormones and lipid profile in patients with HT.

**Methods:**

This randomised controlled clinical trial was conducted on 57 patients with HT. Patients were randomly assigned to receive either an anti‐inflammatory diet plus 1320 mg/day curcumin or an anti‐inflammatory diet plus placebo for 12 weeks. Anthropometric indices, anti‐thyroid peroxidase (anti‐TPO), thyroid‐stimulating hormone (TSH), thyroxine (T4), triiodothyronine (T3), and lipid profile parameters were assessed at baseline and after the 12‐week intervention. The trial was registered in the Clinical Trials Database (registration number NCT05975866).

**Results:**

After 12 weeks of intervention, both groups showed reductions in waist circumference and waist‐to‐hip ratio, with greater changes observed in the curcumin group. However, between‐group differences were not statistically significant. A significant reduction in anti‐TPO levels was observed in the curcumin group compared to placebo (*p* = 0.006). Although TSH and T3 levels significantly decreased within the curcumin group (*p* = 0.014 and *p* = 0.001, respectively), between‐group differences were not statistically significant after adjustment. Additionally, HDL‐C levels showed a non‐significant trend toward improvement in the curcumin group (*p* = 0.053), whereas other lipid parameters remained unchanged.

**Conclusion:**

Curcumin may have possible benefits for thyroid autoimmunity, but further studies are required before any clinical use.

**Trial Registration:**

The trial was registered in the Clinical Trials Database (Registration number: NCT05975866, 08 August 2023). National Nutrition and Food Technology Research Institute NCT0597586 https://www.clinicaltrials.gov/study/NCT05975866?term=NCT05975866&rank=1

## Graphical abstract

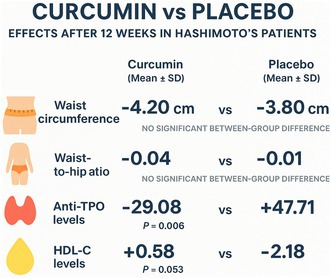

## 1 Introduction

Hashimoto's thyroiditis (HT), also known as chronic lymphocytic thyroiditis, is the most common autoimmune disorder affecting the thyroid gland [1]. It has a global prevalence of 10%–12% in adults and is diagnosed more frequently in women than in men, with annual incidence rates of 350 and 80 per 100,000 people, respectively [2]. HT impairs thyroid function, leading to symptoms such as chronic fatigue, mood swings, and cardiovascular or digestive issues [3]. In the development of HT, genetic factors contribute to 70%–80% of the risk, while environmental factors account for 20%–30% [1]. Many patients with HT, even those with subclinical hypothyroidism, are overweight and exhibit chronic inflammation [4].

Dietary components are reported to influence the health status of patients with HT [5]. Although further research is needed, current evidence suggests that following an anti‐inflammatory diet may be beneficial for preventing the progression of HT [6]. In this context, the anti‐inflammatory effects of several dietary micronutrients, including selenium and vitamin D, have been investigated in patients with HT [7, 8]. Moreover, curcumin (diferuloylmethane), a polyphenol compound and the active ingredient of turmeric ( *Curcuma longa* ), has been extensively studied for its potential health benefits [9]. In recent decades, it has received significant attention for its therapeutic properties, including anti‐inflammatory, anti‐diabetic, anti‐cancer, and anti‐aging effects [10]. Additionally, curcumin has been shown to have a protective effect against changes induced in the thyroid gland by sodium fluoride in albino rats [11]. Recent studies suggest that curcumin's anti‐inflammatory effects may be enhanced when combined with diets that reduce systemic inflammation [12]. An anti‐inflammatory diet can modulate gut microbiota and reduce oxidative stress, which are both implicated in autoimmune thyroid disorders [13, 14]. Since curcumin has poor bioavailability on its own, its therapeutic potential may be amplified when consumed alongside anti‐inflammatory foods that support absorption and synergistic action [9]. Therefore, combining curcumin supplementation with an anti‐inflammatory diet may offer a more comprehensive approach to managing inflammation and improving thyroid function in patients with HT.

Long‐term treatment of HT often requires lifelong levothyroxine therapy [3]. Emerging evidence highlights that dietary modifications may improve thyroid function and the quality of life in patients with HT [10]. Given the increasing prevalence of HT [15] and the critical metabolic role of thyroid hormones, as well as the limited human studies on the effects of curcumin, further research on curcumin's effects is warranted. Therefore, this clinical trial aimed to evaluate the combined effects of an anti‐inflammatory diet and curcumin supplementation on anthropometric measures, thyroid hormones, anti‐thyroid peroxidase (anti‐TPO) levels, and lipid profiles in patients with HT.

## 2 Methods and Materials

### 2.1 Study Design and Participants

This randomised controlled clinical trial was conducted on patients with HT referred to centers affiliated with Shahid Beheshti University of Medical Sciences in Tehran, Iran, during 2023–2024. The trial was registered at ClinicalTrials.gov (ID: NCT05975866, 08 August 2023). Eligible patients, based on laboratory findings and confirmed by endocrinologist approval, were invited to participate. Inclusion criteria included willingness to participate, a confirmed diagnosis of HT by an endocrinologist, age between 20 and 70 years, and a body mass index (BMI) between 18.5 and 35 kg/m^2^. Participants were excluded if they were receiving glucocorticoids, non‐steroidal anti‐inflammatory drugs, or carvedilol. Further exclusion criteria comprised pregnancy or breastfeeding, kidney disease, adherence to a weight‐loss diet within the 3 months preceding enrollment, and the use of weight‐loss medications, fibre preparations, or herbal, vitamin, and mineral supplements. Prior to enrollment, written informed consent was obtained from all participants. The study protocol was approved by the ethics committee of Shahid Beheshti University of Medical Sciences, Tehran, Iran (IR.SBMU.NNFTRI.REC.1402.035).

Sample size was calculated based on TSH as the dependent variable, ensuring a minimum detectable difference of 4 mg/L between groups with 95% confidence (*α* = 0.05) and 80% power (*β* = 0.20). Required number of participants for each group was estimated to be 26 patients; considering the potential loss to follow‐up, 31 patients were planned to be included in each group. Stratified block randomization was applied to evenly distribute participants based on levothyroxine intake and gender. Random allocation of patients was performed by a third party. Throughout the intervention, researchers and participants were blinded to the group allocation. Envelopes were opened sequentially to assign participants to groups. Participants were monitored weekly via phone calls to ensure adherence and address concerns. Additionally, three 24‐h dietary recalls were obtained at baseline, week 6, and week 12 to assess compliance. Participants who deviated from the prescribed diet or supplement by more than 10% were excluded from the final analysis.

Of the 84 patients assessed for eligibility, 18 did not participate in the study due to not meeting the inclusion criteria and four due to unwillingness. Sixty‐two eligible participants met the inclusion criteria and were randomly assigned to either the curcumin group (1320 mg of curcumin daily: three 440 mg capsules with each main meal) (*n* = 31) or the placebo group (*n* = 31) (three 440 mg placebo capsules containing microcrystalline cellulose, taken with each main meal) [16]. The intervention spanned 12 weeks, during which participants received either curcumin or placebo capsules (Arjuna Natural Extracts Ltd., Kerala, India) alongside a prescribed anti‐inflammatory diet. Each curcumin capsule contained 440 mg of curcuminoids (347 mg curcumin, 84 mg desmethoxycurcumin, and 9 mg bis‐desmethoxycurcumin) along with 38 mg of turmeric oil. The placebo capsule had the same shape and colour as the curcumin capsule and contained 444 mg of cooked rice flour. Both groups followed an anti‐inflammatory diet designed based on a structured food composition table (Appendix S1), emphasizing foods rich in bioactive compounds with anti‐inflammatory properties. Participants consumed meals containing whole grains (e.g., rye and whole grain bread), olive oil, berries (raspberry, blackberry), nuts (almonds, hazelnuts), legumes (lentils, red beans), vegetables (tomato, carrot, eggplant, artichoke), and fatty fish (e.g., salmon). These foods provided key anti‐inflammatory compounds such as ferulic acid, oleocanthal, apigenin, luteolin, gallic acid, epicatechin, quercetin, kaempferol, catechin, lycopene, genistein, caffeic acid, resveratrol, and omega‐3—polyunsaturated fatty acids (PUFAs). Daily menus were standardized and monitored to ensure consistent intake of these compounds across participants.

### 2.2 Data Gathering

Dietary intake and physical activity were assessed through face‐to‐face interviews using three 24‐h dietary recalls and a validated International Physical Activity Questionnaire (IPAQ) [17], respectively. Anthropometric parameters, including weight, height, waist circumference, and hip circumference, were measured at baseline and after 12 weeks. Weight and height were assessed using a digital electronic weighing scale (Seca 707; Seca, Hanover, MD) with a precision of up to 100 g, and a tape meter stadiometer, respectively. Waist circumference was measured at the midpoint between the lower margin of the last palpable rib and the top of the iliac crest using a stretch‐resistant tape, while hip circumference was measured at the widest point over the greater trochanters. BMI was calculated as weight (kg) divided by the square of height (m^2^), and the waist‐to‐hip ratio (WHR) was calculated as waist circumference (cm) divided by hip circumference (cm).

### 2.3 Biochemical Measurements

A 10 mL venous blood sample was collected at baseline and at the end of 12 weeks, following a fasting period of 12–14 h. Serum was separated and stored at −80°C for biochemical analysis. Serum levels of T3, T4, and TSH were measured using enzyme‐linked immunosorbent assay (ELISA) kits, following the manufacturer's specifications (Pishtaz Teb Company, Tehran, Iran). Serum anti‐TPO levels were assessed using an anti‐TPO Monokit ELISA kit (Saman Tajhiz Noor Company, Tehran, Iran). Lipid profile levels, including total cholesterol (TC), triglycerides (TG), high‐density lipoprotein cholesterol (HDL‐C), and low‐density lipoprotein cholesterol (LDL‐C), were measured using a standard kit manufactured by Adit brand (Delta Darman Company, Tehran, Iran).

### 2.4 Statistical Analyses

Statistical analyses were conducted using SPSS version 24 (SPSS Inc., Chicago, IL, USA). Quantitative and qualitative data are presented as mean ± standard deviation and frequency (percentage), respectively. Data normality was assessed using the Kolmogorov–Smirnov test. Given the normal distribution of the data and the equality of their variances, the independent‐samples *t*‐test was used to compare the means between groups, while the paired‐samples *t*‐test was used to evaluate changes within the groups over time. The chi‐square test was employed to compare qualitative variables between the groups. Analysis of covariance (ANCOVA) was applied to adjust for confounding factors, including baseline values of the variables. A *p*‐value < 0.05 was considered statistically significant.

## 3 Results

**FIGURE 1:**
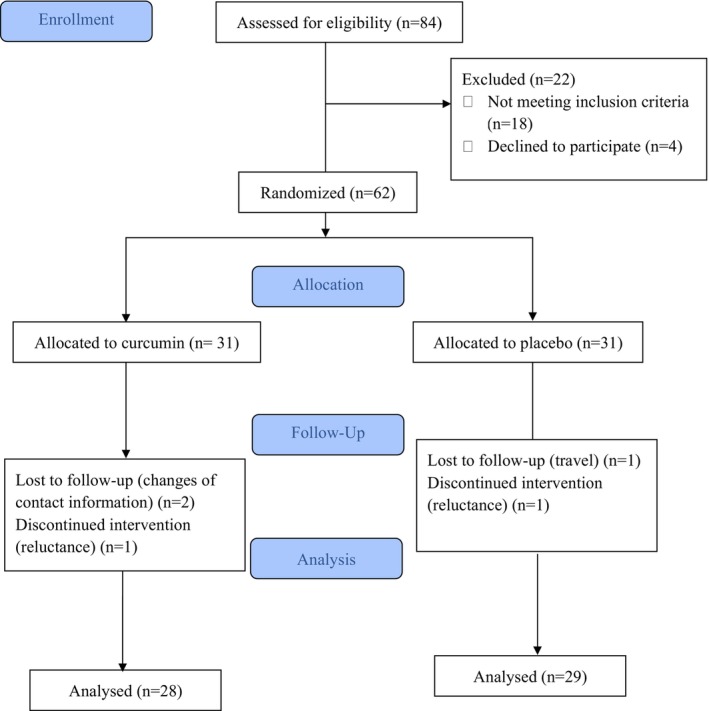
Flowchart of the study participants.

During the 12‐week intervention, three participants from the curcumin group and two from the placebo group withdrew from the study. Final analysis was conducted on 57 patients: 28 in the curcumin group and 29 in the placebo group (Figure 1). Baseline characteristics of participants are presented in Table 1, with no significant differences observed between groups.

**TABLE 1:** Baseline characteristics of Hashimoto's patients in curcumin and placebo groups.

Variable	Curcumin group (*n* = 28)	Placebo group (*n* = 29)	*p* ^a^
Gender (female, %)	89.3%	86.2%	0.723
Age (years)	42.14 ± 12.63	44.59 ± 9.88	0.419
Duration of Hashimoto's (years)	7.22 ± 5.50	7.19 ± 6.20	0.378
Thyroid medication use (%)	71.4%	72.4%	0.934
Marital status (married, %)	64.3%	72.4%	0.631
Smoking (%)	10.7%	20.7%	0.302
Hookah use (%)	25.0%	17.2%	0.473
Alcohol use (%)	21.4%	13.8%	0.449
Physical activity (MET‐min/day)	39.34 ± 3.11	38.59 ± 3.65	0.491

*Note:* Values are presented as mean ± SD for continuous variables and percentages for categorical variables.

^a^ Independent sample *t*‐test for continuous variables and chi‐square test for categorical variables.

As shown in Table 2, both groups experienced reductions in waist circumference and waist‐to‐hip ratio, with greater changes in the curcumin group. Specifically, the curcumin group showed a significant reduction in waist circumference (−4.20 cm, *p* = 0.042) and waist‐to‐hip ratio (−0.04, *p* = 0.038) after 12 weeks of intervention. However, between‐group comparisons for these measures were not statistically significant (*p* = 0.550 and *p* = 0.204, respectively). No significant differences were found between groups regarding changes in weight, BMI, or hip circumference.

**TABLE 2:** Comparison of anthropometric changes between curcumin and placebo groups after 12 weeks.

Variable	Time point	Curcumin group (mean ± SD)	Placebo group (mean ± SD)	Between‐group *p* ^a^
Height (cm)	—	164.20 ± 7.98	163.50 ± 6.63	0.721
Weight (kg)	Start	78.76 ± 13.51	80.13 ± 12.53	0.409
	End	78.60 ± 12.82	79.60 ± 11.37	
	*p*‐value (within‐group)	0.329	0.069	
BMI (kg/m^2^)	Start	29.21 ± 4.64	30.10 ± 5.33	0.424
	End	29.16 ± 4.13	29.84 ± 4.77	
	*p*‐value (within‐group)	0.353	0.091	
Waist circumference (cm)	Start	98.73 ± 8.35	101.31 ± 8.63	0.550
	End	94.53 ± 12.42	97.11 ± 11.99	
	*p*‐value (within‐group)	0.042	0.134	
Hip circumference (cm)	Start	109.69 ± 7.68	112.08 ± 7.33	0.184
	End	109.42 ± 9.05	108.89 ± 8.06	
	*p*‐value (within‐group)	0.935	0.115	
Waist‐to‐hip ratio	Start	0.90 ± 0.06	0.90 ± 0.07	0.204
	End	0.86 ± 0.08	0.89 ± 0.09	
	*p*‐value (within‐group)	0.038	0.705	

*Note:* Values are presented as mean changes ± standard deviation. Height was not included in the analysis as it remained unchanged.

^a^ Independent *t*‐test for between‐group comparison.

Table 3 summarises dietary intake changes. Both groups showed reductions in carbohydrate intake (−42.94 ± 91.45 g/day in curcumin, *p* = 0.027; −37.11 ± 79.44 g/day in placebo, *p* = 0.035) and polyunsaturated fat intake (−2.81 ± 4.29 g/day in curcumin, *p* = 0.003; −3.30 ± 6.87 g/day in placebo, *p* = 0.031). Fibre intake decreased significantly only in the placebo group (−8.81 ± 11.73 g/day, *p* = 0.002). However, none of the between‐group differences in dietary intake was statistically significant.

**TABLE 3:** Comparison of dietary intake changes between curcumin and Placebo groups after 12 weeks.

Variable	Time point	Curcumin group (mean ± SD)	Placebo group (mean ± SD)	Between‐group *p* ^a^
Energy (kcal/d)	Start	1669.52 ± 751.04	1510.39 ± 457.41	0.814
	End	1431.98 ± 552.36	1283.68 ± 500.39	
	*p*‐value (within‐group)	0.065	0.097	
Protein (g/d)	Start	83.43 ± 53.25	70.77 ± 26.20	0.847
	End	78.08 ± 36.67	64.09 ± 23.58	
	*p*‐value (within‐group)	0.467	0.413	
Carbohydrates (g/d)	Start	223.32 ± 94.81	198.96 ± 68.68	0.816
	End	184.94 ± 49.15	165.99 ± 63.72	
	*p*‐value (within‐group)	0.027	0.035	
Fat (g/d)	Start	51.33 ± 29.41	51.69 ± 28.62	0.898
	End	47.00 ± 29.94	46.63 ± 30.43	
	*p*‐value (within‐group)	0.337	0.540	
Cholesterol (mg/d)	Start	301.66 ± 314.81	281.93 ± 232.20	0.425
	End	272.22 ± 265.90	294.01 ± 221.14	
	*p*‐value (within‐group)	0.496	0.662	
Saturated Fat (g/d)	Start	16.90 ± 7.85	17.62 ± 11.38	0.988
	End	15.71 ± 12.56	15.59 ± 10.45	
	*p*‐value (within‐group)	0.530	0.578	
Monounsaturated Fat (g/d)	Start	15.09 ± 8.00	14.85 ± 11.13	0.762
	End	13.21 ± 10.75	11.12 ± 5.65	
	*p*‐value (within‐group)	0.266	0.180	
Polyunsaturated Fat (g/d)	Start	8.05 ± 4.27	7.42 ± 6.10	0.766
	End	5.62 ± 4.00	4.43 ± 2.56	
	*p*‐value (within‐group)	0.003	0.031	
Fibre Intake (g/d)	Start	20.18 ± 12.11	20.56 ± 10.93	0.366
	End	15.36 ± 9.22	11.56 ± 6.13	
	*p*‐value (within‐group)	0.126	0.002	
Carotenoids Intake (μg/d)	Start	3249.92 ± 3095.28	3595.44 ± 3301.09	0.702
	End	2375.84 ± 3049.61	3191.45 ± 3990.48	
	*p*‐value (within‐group)	0.211	0.857	
Selenium (μg/d)	Start	91.97 ± 72.26	82.96 ± 37.75	0.831
	End	68.62 ± 44.04	62.85 ± 24.88	
	*p*‐value (within‐group)	0.060	0.055	
Zinc (mg/d)	Start	13.20 ± 10.11	10.27 ± 4.10	0.536
	End	10.22 ± 7.35	8.12 ± 3.35	
	*p*‐value (within‐group)	0.110	0.131	
Vitamin A (μg/d)	Start	218.21 ± 64.82	300.07 ± 214.29	0.614
	End	229.57 ± 154.69	260.49 ± 265.84	
	*p*‐value (within‐group)	0.829	0.655	
Vitamin E (mg/d)	Start	4.03 ± 3.46	3.56 ± 2.22	0.760
	End	2.72 ± 1.56	2.72 ± 1.34	
	*p*‐value (within‐group)	0.058	0.118	
Vitamin C (mg/d)	Start	54.93 ± 68.70	64.42 ± 77.48	0.868
	End	30.65 ± 33.65	28.33 ± 36.12	
	*p*‐value (within‐group)	0.060	0.074	
Copper (mg/d)	Start	1.17 ± 1.74	1.02 ± 0.56	0.961
	End	0.87 ± 0.45	0.69 ± 0.25	
	*p*‐value (within‐group)	0.052	0.079	

*Note:* Values are presented as mean changes ± standard deviation.

^a^ Independent *t*‐test for between‐group comparison.

Changes in lipid profile are presented in Table 4. HDL‐C levels showed a non‐significant trend toward improvement in the curcumin group (+0.29 ± 4.26 mg/dL, *p* = 0.721) compared to a significant decrease in the placebo group (−2.17 ± 5.49 mg/dL, *p* = 0.042). The between‐group comparison for HDL‐C approached significance (*p* = 0.053). Other lipid parameters, including total cholesterol, triglycerides, and LDL‐C, did not differ significantly between groups.

**TABLE 4:** Comparison of lipid profile changes between curcumin and Placebo groups after 12 weeks.

Variable	Time point	Curcumin group (mean ± SD)	Placebo group (mean ± SD)	Between‐group *p* ^a^	Adjusted *p* ^b^
TC (mg/dL)	Start	160.00 ± 39.07	164.20 ± 37.09	0.278	0.170
	End	149.55 ± 29.80	160.89 ± 38.94		
	*p*‐value (within‐group)	0.105	0.392		
TG (mg/dL)	Start	126.00 ± 49.76	135.51 ± 64.11	0.984	0.826
	End	125.37 ± 54.36	135.20 ± 67.08		
	*p*‐value (within‐group)	0.961	0.932		
LDL‐C (mg/dL)	Start	71.85 ± 18.51	83.06 ± 24.21	0.156	0.444
	End	73.77 ± 20.32	79.00 ± 21.07		
	*p*‐value (within‐group)	0.558	0.184		
HDL‐C (mg/dL)	Start	37.60 ± 5.82	38.00 ± 8.46	0.067	0.053
	End	38.18 ± 7.03	35.82 ± 7.11		
	*p*‐value (within‐group)	0.721	0.042		

*Note:* Values are presented as mean changes ± standard deviation.

^a^ Independent *t*‐test for between‐group comparison.

^b^ Adjusted for baseline values and zinc intake.

Thyroid hormone changes are shown in Table 5. Curcumin supplementation was associated with reductions in T3 (−0.34 ± 0.49 ng/mL, *p* = 0.001), TSH (−2.38 ± 4.69 mIU/L, *p* = 0.014), and anti‐TPO (−33.71 ± 110.61 IU/mL, *p* = 0.015) compared to the baseline. In the placebo group, only T3 showed a significant reduction (−0.18 ± 0.36 ng/mL, *p* = 0.011). Between‐group comparison was statistically significant only for anti‐TPO levels (*p* = 0.006). The difference in TSH changes between groups was marginal (*p* = 0.056) and became non‐significant after adjustment for baseline values.

**TABLE 5:** Comparison of thyroid hormone changes between curcumin and Placebo groups after 12 weeks.

Variable	Time point	Curcumin group (mean ± SD)	Placebo group (mean ± SD)	Between‐group *p* ^a^	Adjusted *p* ^b^
T4 (mg/L)	Start	8.04 ± 1.60	8.70 ± 1.53	0.782	0.347
	End	7.94 ± 1.45	8.77 ± 2.05		
	*p*‐value (within‐group)	0.878	0.806		
T3 (mg/L)	Start	1.85 ± 0.62	1.81 ± 0.51	0.175	0.193
	End	1.54 ± 0.44	1.63 ± 0.48		
	*p*‐value (within‐group)	0.001	0.011		
TSH (mg/L)	Start	4.39 ± 4.61	3.01 ± 3.13	0.056	0.186
	End	2.15 ± 1.68	2.51 ± 2.27		
	*p*‐value (within‐group)	0.014	0.221		
Anti‐TPO (mg/L)	Start	166.91 ± 195.00	150.10 ± 148.26	0.005	0.006
	End	137.83 ± 151.92	197.81 ± 188.24		
	*p*‐value (within‐group)	0.015	0.125		

*Note:* Values are presented as mean changes ± standard deviation.

^a^ Independent *t*‐test for between‐group comparison.

^b^ Adjusted for baseline values and zinc intake.

To assess the robustness of the findings and account for potential gender‐related bias, a sensitivity analysis was conducted by excluding male participants. The results among female patients remained consistent with the primary analysis. Specifically, curcumin supplementation led to a greater reduction in serum anti‐TPO levels compared to placebo (adjusted *p* = 0.010), while no significant between‐group differences were observed for T3 (adjusted *p* = 0.162), T4 (adjusted *p* = 0.494), or TSH (adjusted *p* = 0.403). These findings are presented in Appendix S2.

## 4 Discussion

To the best of our knowledge, this study represents the first clinical trial investigating the combined effects of an anti‐inflammatory diet and curcumin supplementation in patients with HT. Anti‐TPO levels, along with waist circumference and waist‐to‐hip ratio, showed notable reductions in the curcumin group compared to placebo, while other thyroid‐related and metabolic markers remained largely unchanged. Curcumin is the key active compound in turmeric [15] and previous studies have demonstrated its anti‐inflammatory properties [18], antioxidant [19, 20], antitumor [21, 22], and immune‐regulating effects [23], along with therapeutic potential in neurodegenerative [24], cardiovascular [25], and cerebrovascular diseases [26]. Our study found that curcumin supplementation had a minimal impact on HDL‐C, while TC, TG, and LDL‐C levels remained unchanged. Similarly, a human trial assessed the effects of curcumin supplementation on blood lipid profiles, specifically examining TC, LDL‐C, HDL‐C, and triglyceride levels. This study included individuals with dyslipidemia, especially those with elevated total cholesterol and triglyceride levels. Participants received 600 mg of curcumin daily for a duration of 6 months. Curcumin showed no significant effects on TC, TG, LDL‐C, or HDL‐C cholesterol over the six‐month period [27, 31].

This discrepancy may be attributed to differences in the participants' baseline lipid profiles, curcumin doses, and intervention durations. It is important to note that our study was conducted in patients with Hashimoto's thyroiditis, not individuals with dyslipidemia or elevated baseline lipid levels. This distinction may partly explain the lack of significant changes in lipid parameters, in contrast to studies targeting hyperlipidemic populations.

Curcumin supplementation (30 mg/kg/day) effectively restored elevated oxidative stress markers, such as lipid peroxidation and protein carbonylation, and replenished antioxidant molecules like glutathione and ascorbic acid. Additionally, curcumin reduced the overexpression of copper/zinc superoxide dismutase (Cu/Zn‐SOD) and manganese superoxide dismutase (Mn‐SOD) enzymes induced by T4 [28]. Another study investigated the protective effects of curcumin against thyroid hormone (TH) imbalances in male rats following traumatic brain injury (TBI) induced by gas explosions. It highlighted that gas explosions can induce pathological changes in the thyroid and alter the hypothalamic–pituitary–thyroid axis, leading to unbalanced serum TH levels. While these animal studies offer valuable mechanistic insights into curcumin's potential effects on thyroid function and oxidative stress pathways, their findings may not fully translate to human physiology. Our trial offers preliminary evidence in a human population, but the findings should be interpreted with caution due to the study's limited size and duration. These results support the need for further human studies to validate and expand upon the therapeutic potential of curcumin in autoimmune thyroid conditions. Furthermore, although the reduction in anti‐TPO levels is promising, it should be interpreted with caution, as antibody titers may fluctuate over time and short‐term changes may not necessarily reflect long‐term disease modification.

Regarding the effects of curcumin on anthropometric indices, the results of the present study indicated that curcumin significantly reduced waist circumference and waist‐to‐hip ratio. Curcumin has gained recognition for its association with the reduction of waist size, especially among obese individuals and those affected by metabolic disorders. Evidence from several studies suggests that the anti‐inflammatory and antioxidant activities of curcumin significantly contribute to improvements in metabolic health, leading to a reduction in waist circumference. A study by Sharif et al. on overweight females demonstrated that participants lost approximately 5 cm in waist circumference after 12 weeks of treatment with curcumin [29]. Similarly, Akbari et al. suggested that curcumin administration led to significant reductions in BMI as well as waist size in individuals with metabolic syndrome [30]. Furthermore, Unhapipatpong et al. demonstrated significant reductions in waist circumference in women with polycystic ovary syndrome [27]. These findings suggest that curcumin may contribute to improvements in body composition, particularly waist circumference, although further studies are needed.

Our study has several strengths, including a randomised controlled trial design, which ensured balanced allocation of participants based on levothyroxine intake and gender between the curcumin and placebo groups, thereby reducing selection bias. The use of a double‐blind, placebo‐controlled method enhanced the reliability of the findings by minimising performance and detection bias. Additionally, adherence was closely monitored through regular follow‐ups, capsule counts, and phone calls to maintain compliance. Strict inclusion and exclusion criteria minimised confounding variables, enhancing the validity of the findings. However, this study has several limitations that should be acknowledged. First, although the sample size was calculated based on TSH as the primary outcome, it may not have been adequately powered to detect subtle changes in lipid profiles or anthropometric measures. This limits the strength of conclusions regarding metabolic outcomes. Second, the 12‐week duration, while sufficient to observe short‐term biochemical changes, is too brief to determine whether the observed effects—particularly reductions in anti‐TPO and TSH—are sustained or clinically meaningful in the long term. Third, both groups received an anti‐inflammatory diet, and dietary adherence was assessed through self‐reported 24‐h recalls, which may introduce recall bias and limit the precision of dietary exposure assessment. The fact that both groups showed reductions in T3 levels suggests that dietary changes may have contributed to the outcomes, potentially confounding the specific effects of curcumin supplementation. Although stratified randomization was applied to balance gender, the small number of male participants prevented meaningful sex‐specific analysis. Future studies should consider longer follow‐up periods, larger and more diverse populations, and factorial or multi‐arm designs to disentangle the independent and combined effects of curcumin and diet. Additionally, providing standardised meals or objective biomarkers of dietary adherence could improve dietary control.

## 5 Conclusion

Curcumin supplementation, when combined with an anti‐inflammatory diet, was associated with reductions in waist circumference, waist‐to‐hip ratio, and serum anti‐TPO levels in patients with Hashimoto's thyroiditis. However, changes in TSH and T3 levels were not significantly different between groups after adjustment, and both groups showed a reduction in T3, suggesting that dietary factors may have contributed to the observed effects. Given the modest changes and short duration of the study, these findings should be interpreted with caution. Further research with larger sample sizes and longer follow‐up periods is warranted to evaluate the potential role of curcumin as an adjunctive strategy in managing autoimmune thyroid conditions.

## Author Contributions

Conceptualization, G.S. and S.K. Formal analysis, Z.Y. Methodology, F.B., B.M., N.N. and S.M. Project administration, F.B., G.S. and S.K. Writing – original draft, F.B., Z.Y. and S.K. Writing – review and editing, Z.Y. and G.S. All authors read and approved.

## Ethics Statement

The study protocol was approved by the ethics review committee of Shahid Beheshti University of Medical Sciences, Tehran, Iran (IR.SBMU.NNFTRI.REC.1402.035) and the research was carried out in accordance with the principles outlined.

## Consent

All participants voluntarily provided written informed consent.

## Conflicts of Interest

The authors declare no conflicts of interest.

## Supporting information


**Appendix S1:** edm270138‐sup‐0001‐AppendixS1.docx.

**Appendix S2:** edm270138‐sup‐0002‐AppendixS2.docx.

## Data Availability

The data that support the findings of this study are available from the corresponding author upon reasonable request.
