# Supplementary material for: The Combined Effects of an Anti‐Inflammatory Diet and Curcumin Supplementation on Thyroid Function and Lipid Profile in Patients With Hashimoto's Thyroiditis: A Double Blind Randomised Clinical Trial
Source: Endocrinol Diabetes Metab. 2025 Dec 2;9(1):e70138. doi: 10.1002/edm2.70138 (PMC12671534; doi:10.1002/edm2.70138)
Supplement: Supplementary file 1 — Appendix S1: edm270138‐sup‐0001‐AppendixS1.docx. [file EDM2-9-e70138-s002.docx]

**Supplementary file 1. Anti-Inflammatory Food Composition Table**

| **Breakfast** | | **Grams** | **Anti-inflammatory compound (mg/100g)** | | **Milligram** s |
| --- | --- | --- | --- | --- | --- |
| **Whole grain bread** | | 60 | 72 | Ferulic acid | 43.20 |
| Olive oil | | 10 | 5.4 | Oleocanthal | 0.54 |
|  |  |  | 1.2 | Apigenin | 0.12 |
|  |  |  | 0.4 | Luteolin | 0.04 |
| Raspberry | | 50 | 4.7 | Gallic acid | 2.35 |
|  |  |  | 12 | Epicatechin | 6.00 |
| Blackberry | | 50 | 1.6 | Resveratrol | 0.80 |
| **Morning snack** | | **Grams** | **Anti-inflammatory compound (mg/100g)** | | **Milligram** s |
| **Pomegranate juice** | | 200 | 44 | Panicalgin | 88.00 |
| Raw almonds | | 30 | 7.2 | Catechin | 2.16 |
| Raw hazelnuts | | 40 | 480 | Gallic acid | 192.00 |
| **Lunch** | | **Grams** | **Anti-inflammatory compound (mg/100g)** | | **Milligram** s |
| **Salad** | **Rhubarb** | 122 | 2.2 | Catechin | 2.68 |
|  | Black olives | 16 | 4.1 | Epicatechin | 0.65 |
|  |  |  | 8.2 | Apigenin | 1.31 |
|  | Green olives | 16 | 3 | Ferulic acid | 0.48 |
|  | Barberry | 8.6 | 33 | Quercetin | 2.83 |
|  |  |  | 104 | Kaempferol | 8.94 |
|  | Tomato | 100 | 4.2 | Lycopene | 4.20 |
|  | Vinegar | 10 | 2.6 | Gallic acid | 0.26 |

|  | Olive oil | 10 | 5.4 | Oleocanthal | 0.54 |
| --- | --- | --- | --- | --- | --- |
|  |  |  | 1.2 | Apigenin | 0.12 |
|  |  |  | 0.4 | Luteolin | 0.04 |
| Lentil and vegetable stew | Lentils | 40 | 0.3 | Catechin | 0.12 |
|  | Red beans | 20 | 0.6 | Genistein | 0.12 |
|  |  |  | 2 | Kaempferol | 0.40 |
|  | Carrot | 50 | 8.3 | Beta-carotene | 4.15 |
|  | Tomato | 90 | 4.2 | Lycopene | 3.78 |
|  | Shallot | 25 | 2 | Quercetin | 0.50 |
|  | Olive oil | 10 | 5.4 | Oleocanthal | 0.54 |
|  |  |  | 1.2 | Apigenin | 0.12 |
|  |  |  | 0.4 | Luteolin | 0.04 |
|  | Pune | 1 | 21 | Caffeic acid | 0.21 |
|  |  |  | 3.3 | Apigenin | 0.03 |
|  |  |  | 5.2 | Gallic acid | 0.05 |
|  | Cumin | 1 | 39 | Kaempferol | 0.39 |
|  | Clove | 1 | 458 | Gallic acid | 4.58 |
| Rye bread | | 60 | 0.8 | Epicatechin | 0.48 |
| Plum | | 200 | 4.6 | Catechin | 9.20 |
| Dark chocolate | | 20 | 25 | Quercetin | 5.00 |
|  |  |  | 0.1 | Resveratrol | 0.02 |
|  |  |  | 21 | Catechin | 4.20 |
|  |  |  | 24 | Ferulic acid | 4.80 |
| **Evening meal** | | **Grams** | **Anti-inflammatory compound (mg/100g)** | | **Milligram** |
| **Orange juice** | | 200 | 0.06 | Tangerine | 0.12 |
| Raw almonds | | 30 | 7.2 | Catechin | 2.16 |

| **Dinner** | | **Grams** | **Anti-inflammatory compound (mg/100g)** | | **Milligram** |
| --- | --- | --- | --- | --- | --- |
| **Grilled fish with vegetables** | **Fatty fish like salmon** | 150 | 2000 | ω-3 PUFAs | 3000.00 |
|  | Olive oil | 10 | 5.4 | Oleocanthal | 0.54 |
|  |  |  | 1.2 | Apigenin | 0.12 |
|  |  |  | 0.4 | Luteolin | 0.04 |
|  | Eggplant | 150 | 3 | Ferulic acid | 4.50 |
|  | Artichoke | 70 | 7.4 | Apigenin | 5.18 |
|  | Rosemary | 1 | 10 | Caffeic acid | 0.10 |
|  |  |  | 2.7 | Catechin | 0.027 |
|  |  |  | 0.6 | Apigenin | 0.006 |
| Rye bread | | 60 | 0.8 | Epicatechin | 0.48 |
| grape | | 200 | 0.4 | Resveratrol | 0.80 |
| Dark chocolate | | 20 | 25 | Quercetin | 5.00 |
|  |  |  | 0.1 | Resveratrol | 0.02 |
|  |  |  | 21 | Catechin | 4.20 |
|  |  |  | 24 | Ferulic acid | 4.80 |
