# Supplementary material for: The Combined Effects of an Anti‐Inflammatory Diet and Curcumin Supplementation on Thyroid Function and Lipid Profile in Patients With Hashimoto's Thyroiditis: A Double Blind Randomised Clinical Trial
Source: Endocrinol Diabetes Metab. 2025 Dec 2;9(1):e70138. doi: 10.1002/edm2.70138 (PMC12671534; doi:10.1002/edm2.70138)
Supplement: Supplementary file 2 — Appendix S2: edm270138‐sup‐0002‐AppendixS2.docx. [file EDM2-9-e70138-s001.docx]

| **Supplemental Table 1.** Comparison of thyroid hormones among female participants | | | | | | |
| --- | --- | --- | --- | --- | --- | --- |
| Variable | Group | Research Time | | | P-value^**^ | Adjusted P-value^***^ |
|  |  | Start of Study | End of Study | Mean Changes |  |  |
| T4  (mg/L) | Curcumin | 8.08 ± 1.65 | 7.99 ± 1.53 | -0.04 ± 1.85 | 0.878 | 0.494 |
|  | Placebo | 8.68 ± 1.58 | 8.83 ± 2.13 | 0.16 ± 1.37 | 0.928 |  |
|  | P-value^*^ | 0.198 |  | 0.680 |  |  |
| T3  (mg/L) | Curcumin | 1.9 ± 0.64 | 1.53 ± 0.47 | -0.41 ± 0.48 | <0.001 | 0.162 |
|  | Placebo | 1.79 ± 0.55 | 1.64 ± 0.5 | -0.15 ± 0.34 | 0.042 |  |
|  | P-value | 0.967 |  | 0.030 |  |  |
| TSH  (mg/L) | Curcumin | 4.07 ± 4.23 | 2.09 ± 1.68 | -2.13 ± 4.64 | 0.034 | 0.403 |
|  | Placebo | 3.08 ± 3.34 | 2.31 ± 2.38 | -0.78 ± 2.07 | 0.073 |  |
|  | P-value | 0.365 |  | 0.190 |  |  |
| Anti-TPO  (mg/L) | Curcumin | 175.82 ± 203.86 | 140.73 ± 159.22 | -40.67 ± 115.23 | 0.097 | 0.010 |
|  | Placebo | 115.87 ± 120.16 | 173.39 ± 186.93 | 57.51 ± 97.65 | 0.007 |  |
|  | P-value | 0.211 |  | 0.002 |  |  |
| Values are means ± SDs  *Independent t-test  ** paired t-test  ***Adjusted for zinc intake and baseline Variable level | | | | | | |
